# A Rapidly Stabilizing Water-Gated Field-Effect Transistor Based on Printed Single-Walled Carbon Nanotubes for Biosensing Applications

**DOI:** 10.1021/acsaelm.1c00332

**Published:** 2021-07-01

**Authors:** Alireza Molazemhosseini, Fabrizio Antonio Viola, Felix J. Berger, Nicolas F. Zorn, Jana Zaumseil, Mario Caironi

**Affiliations:** † Center for Nano Science and Technology @PoliMi, Istituto Italiano di Tecnologia, Via Giovanni Pascoli, 70/3, 20133 Milano, Italy; ‡ Institute for Physical Chemistry and Centre for Advanced Materials, Universitaẗ Heidelberg, D-69120 Heidelberg, Germany

**Keywords:** electrolyte-gated, field-effect transistors, semiconducting carbon nanotubes, biosensors, bioelectronics, biotin, streptavidin

## Abstract

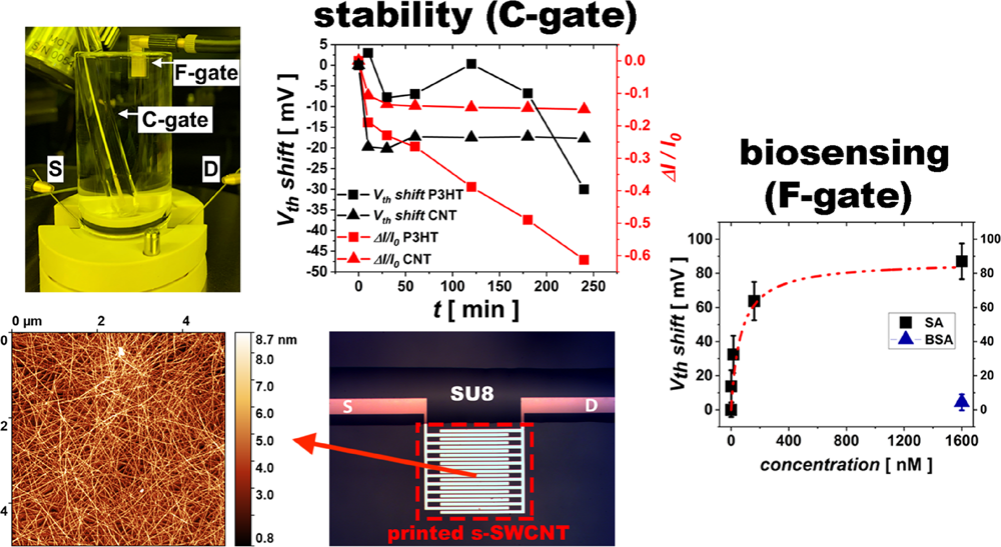

Biosensors are expected to revolutionize disease management through provision of low-cost diagnostic platforms for molecular and pathogenic detection with high sensitivity and short response time. In this context, there has been an ever-increasing interest in using electrolyte-gated field-effect transistors (EG-FETs) for biosensing applications owing to their expanding potential of being employed for label-free detection of a broad range of biomarkers with high selectivity and sensitivity while operating at sub-volt working potentials. Although organic semiconductors have been widely utilized as the channel in EG-FETs, primarily due to their compatibility with cost-effective low-temperature solution-processing fabrication techniques, alternative carbon-based platforms have the potential to provide similar advantages with improved electronic performances. Here, we propose the use of inkjet-printed polymer-wrapped monochiral single-walled carbon nanotubes (s-SWCNTs) for the channel of EG-FETs in an aqueous environment. In particular, we show that our EG-CNTFETs require only an hour of stabilization before producing a highly stable response suitable for biosensing, with a drastic time reduction with respect to the most exploited organic semiconductor for biosensors. As a proof-of-principle, we successfully employed our water-gated device to detect the well-known biotin–streptavidin binding event.

## 1 Introduction

Recently, a lot of efforts have been devoted to the development of new classes of biosensors characterized by features such as a small form factor (i.e., handheld), low-manufacturing costs, and fast response time, aiming at point-of-care systems and self-testing platforms.

Among the different types of transducers used for biosensing, a field-effect transistor (FET) has become an attractive candidate due to its intrinsic signal amplification, which results in a very high sensitivity. A variety of sensing platforms have been developed and extensively studied based on FETs such as ion-sensitive FETs,^1^ floating-gate FETs,^2,3^ and, more recently, electrolyte-gated FETs (EG-FETs),^4^ which have a remarkable potential yet to be fully exploited. In a typical EG-FET, an ionically conducting and electronically insulating electrolyte is employed as a gate insulator. Upon polarization of the gate, ion redistribution occurs, resulting typically in the formation of the so-called electric double layers (EDL), characterized by high capacitance values (>1 μF/cm^2^).^5^ As a consequence, EG-FETs are characterized by low operating voltages (less than 1 V). Moreover, they do not require a reference electrode, which is needed, for instance, in ion-sensitive FETs, and offer label-free detection. In EG-FET biosensors, either the gate or the semiconductor can be biofunctionalized with a specific recognition element.^6^ Although introducing a bioreceptor into the semiconductor could become challenging, gate functionalization has shown to be very robust and versatile owing to the high affinity of thiol-ending molecules to gold gates and to the experimental convenience of thiol functionalization procedures.^7−9^

The quest for low manufacturing costs of EG-FET-based biosensors has made solution-processed organic semiconductors a promising choice for the active material,^10,11^ given their compatibility with large-area and low-temperature printing techniques and with flexible, low-cost substrates.^12−14^ In organic electrolyte-gated transistors, a distinction can be made between the case in which the semiconductor is not permeable to ions in the electrolyte so that an EDL forms at this interface and the case where a volumetric capacitance develops as a result of ion permeation in the semiconductor thin film. In the latter case, the device is historically addressed as an organic electrochemical transistor (OECT) and exploits a volumetric field-effect. Despite the larger capacitance typically displayed by OECTs, the highest sensitivity has been reported so far for biosensors based on the former architecture, which typically goes under the name of electrolyte-gated organic field-effect transistor (EGOFET). In fact, Torsi and collaborators recently reported poly(3-hexylthiophene) (P3HT)-based EGOFET biosensors with a record sensitivity for millimeter-sized devices, down to single molecules (in the zM range), coupled to an excellent selectivity.^4,15−17^

The ultimate sensitivity reachable with biosensors based on solution-processed water-gated polymer FETs and their compatibility with high-throughput manufacturing make the research toward real-world applicable printed EG-FET biosensing platforms extremely appealing.

As a matter of fact, the large majority of water-gated OFETs exploiting a solution-processed active layer make use of spin-coated films, while very few reports can be found on using printing techniques for deposition of the organic semiconductor channel.^18,19^

It is also a common trait of some of the most adopted organic semiconductors in water-gated OFETs to undergo long stabilization times in water before producing a stable response suitable for biosensing. For instance, in the case of P3HT EGOFETs, more than 12 h of water immersion is required.^3,18,20−22^ This process is attributed to a mild polymer swelling when it is in direct contact with water, as demonstrated by the presence of hydroxyl moieties in the polymer film as well as its increased roughness after exposure to water.^23^ A similar behavior was also reported for EGOFETs that use organic semiconductors blended with polystyrene as their active layer.^24^ Indeed, such a conditioning procedure imposes a constraint for the actual use of the biosensor.

In this respect, exploring alternative semiconductors that can be printed and share the same advantages of conjugated organic semiconductors in terms of manufacturing while offering advantages in terms of electronic properties and stability under operation in water is very desirable. A powerful alternative is represented by semiconducting carbon nanotubes (CNTs).^25−30^ With recent advancements in sorting CNTs through selective polymer-wrapping techniques, printable semiconducting single-walled carbon nanotube (s-SWCNT) formulations with high purity have become available.^31,32^ We have previously reported the successful adoption of spin-coated random networks of polymer-wrapped s-SWCNTs in electrolyte-gated carbon nanotube FETs (EG-CNTFETs) for cell proliferation monitoring in aqueous cell culture media.^33^ In this work, we present high-performance EG-FETs operating in water based on inkjet-printed random networks of highly pure polymer-wrapped monochiral (6,5) s-SWCNTs. Our EG-CNTFET shows a normalized transconductance (*g*′_m_ = *g*_m_ × *L*/*W*, *L* being the channel length and *W* the channel width) of 0.44 μS, which is at least one order of magnitude higher compared to its EGOFET counterparts.^4,24^ Importantly, the device shows fast conditioning in water, producing a stable current after only 1 h of consecutive acquisition of transfer curves, thus largely reducing the time constraints on its actual use as a sensor. In order to produce a proof-of-principle of its use as a base for future biosensors, we successfully adopted our inkjet-printed EG-CNTFET to characterize the well-known binding event of biotin to streptavidin.^34−36^ The inkjet-printed EG-CNTFET device presented in this study provides a stable and versatile platform that can be further exploited for the development of arrays of printed biosensors.

## 2 Experimental Section

### 2.1 Preparation of s-SWCNT Dispersions

(6,5) s-SWCNT dispersions were prepared from CoMoCAT carbon nanotubes (Chasm Advanced Materials, SG65i-L58). According to the supplier, this CoMoCAT material has a maximum carbon content of 95%, and 93% of which are SWCNTs. Forty percent of the nanotubes are (6,5) SWCNTs, therefore ∼35 wt % of the raw material. Shear force mixing (SFM) was used for sorting nanotubes through polymer-wrapping using poly[(9,9-dioctylfluorenyl-2,7-diyl)-*alt*-*co*-(6,6′-(2,2′-bipyridine))] (PFO-BPy).^31^ Briefly, 0.5 g/L PFO-BPy (American Dye Source, *M*_W_ = 40 kg/mol) was dissolved in toluene before adding the 0.38 g/L CoMoCAT raw material. SFM using a Silverson L2/Air mixer was then applied at maximum speed (10,230 rpm) for 72 h. The temperature was kept constant at 20 °C with a cooling bath. Next, the dispersion was centrifuged twice for 45 min at 60,000*g* and the supernatant was passed through a poly(tetrafluoroethylene) (PTFE) syringe filter (pore size, 5 μm) to remove undispersed materials and aggregates. To collect the SWCNTs, the resulting dispersion was filtered through a PTFE membrane (Merck Millipore, JVWP, 0.1 μm pore size) and washed with toluene in order to remove excess polymer. The nanotube ink was prepared through redispersion of the s-SWCNT filter cake in 1,2-dichlorobenzene by 30 min of bath sonication, obtaining an optical density of 1 cm^–1^ at the E_11_ transition.

### 2.2 FET Fabrication

**Figure 1:**
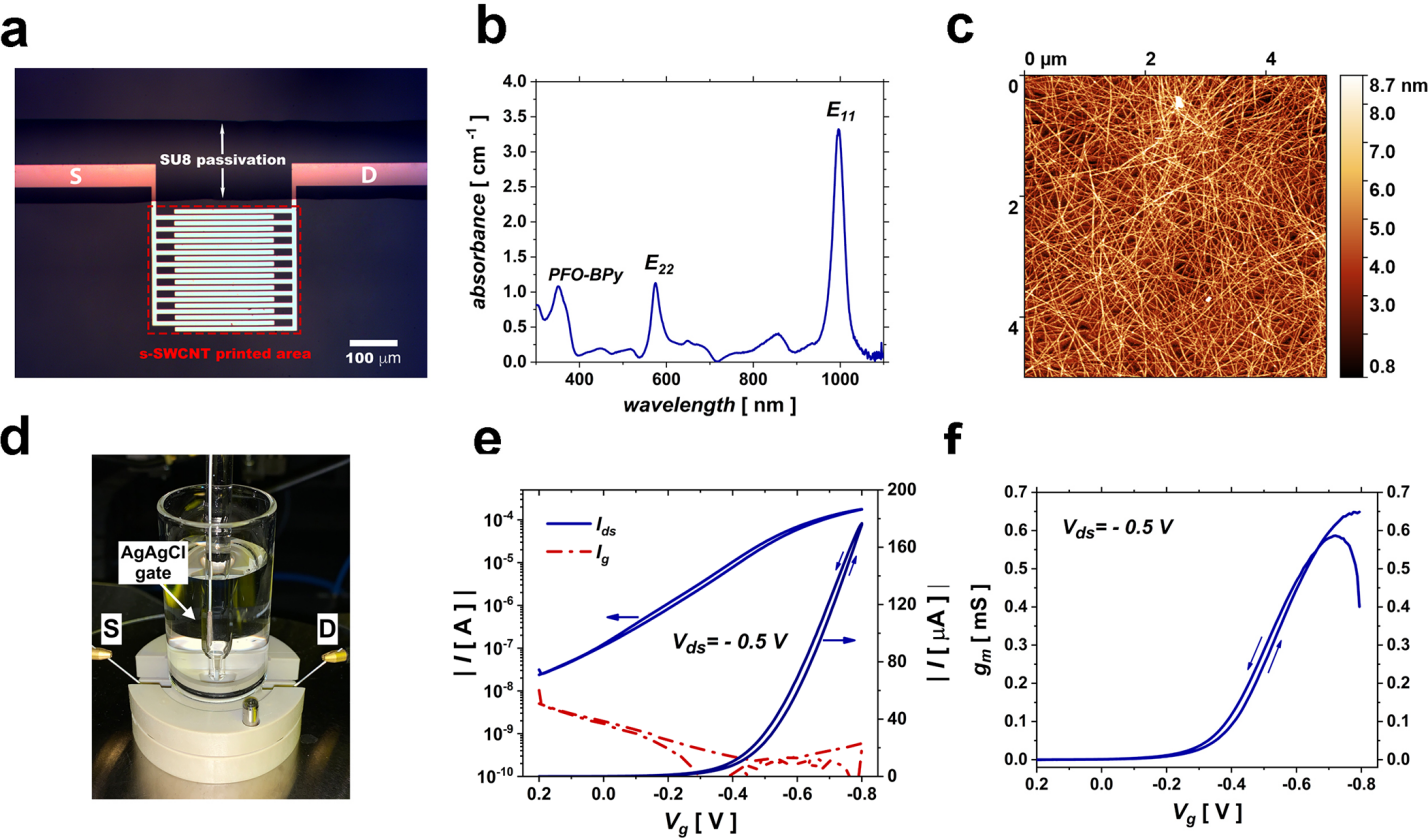
(a) Optical microscope image of the sensing area of the device showing the printed s-SWCNT area (dashed red rectangle, nanotubes are not visible under an optical microscope) onto the interdigitated electrodes and passivated contacts with printed SU8. (b) Absorption spectrum of the (6,5) s-SWCNT ink prepared for printing. (c) AFM topography of a typical inkjet-printed random network of nanotubes. (d) Testing setup used for electrical characterization of the device, consisting of a glass reservoir for accommodating the electrolyte, water in this case, a magnet mount cell at the bottom to sandwich the chip with a circular opening (diameter of 6 mm) to expose the device to the electrolyte, and a Ag/AgCl reference electrode immersed in the electrolyte, used as a gate. (e) Typical transfer characteristic curve of the EG-CNTFET in semilogarithmic (left axis) and linear (right axis) scales showing the drain current in blue together with the gate leakage current in dashed dotted red line, which is five orders of magnitude lower than drain current at *V*_g_ = −0.8 V. (f) Transconductance of the EG-CNTFET calculated from the transfer curve in (e).

Interdigitated source–drain electrodes with *W* = 4.4 mm and *L* = 3 μm were patterned on a glass substrate by a mask-less reverse lithography process (AZ5214 photoresist together with an MLA100 Heidelberg mask-less aligner) including thermal evaporation of chromium (2 nm) and gold (15 nm) followed by lift-off in *N*-methyl-2-pyrrolidone. Gold electrodes were subjected to 3 min of oxygen plasma treatment before inkjet printing. The s-SWCNT ink was inkjet-printed (Fujifilm Dimatix, DMP2831) onto the active area of the patterned electrodes (an area of 300 × 300 μm^2^) with a drop-spacing of 60 μm. Printed patterns were annealed for 5 min at 200 °C in air, rinsed with tetrahydrofuran and isopropanol, and dried with a nitrogen flux. The gate electrode was fabricated by thermally evaporating a Cr (2 nm)/Au (50 nm) stack on a polyethylene naphthalate (PEN) sheet with a defined area of 3 × 3 mm^2^. A film of inkjet-printed biocompatible insulator (SU8 – TF6001 MicroChem) was employed to selectively passivate source and drain electrodes (Figure 1a) as well as to confine the sensing area of the gate electrodes adopted for biosensing.

### 2.3 Surface Biofunctionalization

Gold electrodes on PEN were rinsed in isopropanol for 5 min by bath sonication followed by drying under a nitrogen flux. To form self-assembled monolayers of cysteamine (Cys-SAM), gold electrodes were immersed in a 100 mM cysteamine (Sigma #30070) solution prepared in 0.01 M phosphate buffer saline (PBS, Sigma Aldrich #P4417) and kept in the dark for 24 h inside a nitrogen glovebox. Subsequently, electrodes were rinsed with copious amount of DI water and were treated for 2 h with a freshly prepared 5 mg/mL solution of sulfosuccinimidyl-6-(biotinamido) hexanoate (Sigma Aldrich NHS-X-biotin #203189) in 0.01 M PBS in air at room temperature (RT) by drop casting 50 μL of the solution onto the surface of the electrodes.

### 2.4 Electrical Characterization of the EG-CNTFET

The electrical characterization of the EG-CNTFET was performed by means of a Keysight B1500A Semiconductor Device Parameter Analyzer. The measurement setup included a Redox.me testing cell, which has a bottom magnet mount cell and a top glass reservoir for accommodating the electrolyte. The device was fixed in the cell, and DI water was used as the gating medium. A standard Ag/AgCl electrochemical electrode was employed as the gate for testing the device. The device was interrogated by consecutive recordings of transfer characteristic curves with a scan rate of 0.025 V/s for 4 h with an interval of 3 min in between the acquisitions, during which the device was at a tri-state. Each curve acquisition lasted for 80 s.

### 2.5 Biosensing Measurement

For the proof-of-principle of biosensing experiments, the same setup used for electrical characterization of the EG-CNTFET was used, adopting the Ag/AgCl electrode as the control gate (C-gate) to monitor the stability of the transistor, while a functionalized gold gate (F-gate) was immersed in the water reservoir and exploited for biosensing tests (Figure S1). Before a biosensing experiment, the device undergoes a conditioning procedure to reach electrical stability. This stability protocol involves interrogation of the device kept in water by consecutive recordings of transfer characteristic curves with 3 min intervals until the threshold voltage (*V*_th_) and the current of the device stabilize. The protocol considers *V*_th_ stable when the rate of variation is equal or lower than 1 mV/h and considers the current stable when the rate of the ratio between its temporal variation (Δ*I*) and its initial value (*I*_0_) reaches 0.002 h^–1^. The C-gate is used to apply the stabilization protocol. The last transfer curve obtained at the end of the stabilization protocol is used as the reference to be compared with a transfer curve acquired on the C-gate immediately after obtaining the calibration curve. This is to validate the device stability during the biosensing measurement. Standard solutions of streptavidin were prepared with concentrations ranging from 1.6 nM to 1.6 μM in 0.01 M PBS for the purpose of acquisition of the calibration curve. Negative control measurements were performed by substituting streptavidin with bovine serum albumin (BSA). The titration curve was acquired by drop casting different concentrations of streptavidin solutions on F-gates and incubating for 20 min at RT. F-gates were then rinsed with DI water and immersed in DI water for 10 min before being mounted one by one on the device to record the transfer response. For each concentration of streptavidin, three physically different F-gates were measured.

### 2.6 s-SWCNT Ink and Film Characterization

Optical characterization of s-SWCNT ink was performed by recording the absorption spectra of the formulation using a PerkinElmer Lambda 1050 spectrometer. The surface topography of the s-SWCNT inkjet-printed random network was measured with an Agilent 5500 atomic force microscope operated in tapping mode.

## 3 Results and Discussion

### 3.1 EG-CNTFET Electrical Properties

First, we aimed at assessing the electrical performance of EG-FETs based on printed random networks of polymer-wrapped monochiral (6,5) s-SWCNTs. The relatively large bandgap of (6,5) s-SWCNTs (≈1.27 eV) compared to semiconducting nanotubes with other chiralities is reported to contribute to low off currents in field-effect transistors.^37^ The sorting process through selective wrapping with PFO-BPy involves shear force mixing, which is a mild dispersion method compared to other techniques such as sonication and introduces less structural damage to the SWCNTs.^31^ Consequently, a larger average length of nanotubes can improve the charge transport properties of a random network. Our EG-CNTFET is fabricated by inkjet-printing of the nanotubes onto interdigitated gold electrodes after brief oxygen plasma treatment. Figure 1a presents an optical microscope image of the active area of the device, showing the interdigitated electrodes and the contact leads passivated with inkjet-printed SU8 in order to prevent their exposure to the electrolyte. Nanotube-printed area, which is not visible under an optical microscope, is indicated with a red box in Figure 1a. The absorption spectrum of the s-SWCNT ink prepared for printing (Figure 1b) evidences the two main absorption peaks (E_11_ and E_22_) of (6,5) nanotubes at 996 nm and at 575 nm, along with the PFO-BPy absorption signature at shorter wavelengths. The ink absorbance indicates the absence of metallic nanotubes within the detection limit and a high degree of monochirality. AFM topography of the inkjet-printed s-SWCNT formulation (Figure 1c) evidences a dense random network of nanotubes. Although a single carbon nanotube (average length of about 1.82 μm^31^) is not long enough to bridge the 3 μm channel gap of the device, the random network allows a charge percolation through multiple pathways between the source and drain.

For electrical characterization, the device is placed in a testing cell (Figure 1d) and is immersed in DI water acting as a gating medium. To obtain the transfer characteristic curves of the EG-CNTFET, a Ag/AgCl electrode immersed in DI water is used as a gate, exploiting its well-known stability in aqueous environments. A typical transfer characteristic curve of the printed EG-CNTFET (Figure 1e) shows good p-type operation, with a channel current reaching 180 μA at a gate voltage (*V*_g_) of −0.8 V, a current on/off ratio higher than 10^3^, and a very low gate leakage current, 5 orders of magnitude lower than the on current (at *V*_g_ = – 0.8 V). The device achieves a maximum transconductance *g*_m_ of ∼645 μs, corresponding to a normalized transconductance (*g*_m_ divided by *W/L*) of 0.44 μS (Figure 1f). Overall, our printed EG-CNTFET demonstrates a current on/off ratio comparable to the best water-gated EGOFET counterparts^4,19^ while showing at least one order of magnitude higher normalized transconductance.^11,38^ Therefore, the proposed water-gated device is both compatible with large-area printing processes and delivers competitive electrical performances.

### 3.2 Stabilization of the EG-CNTFET in Water

A stable device response upon exposure to the electrolyte is crucial for biosensing applications. No sensitive biosensing is possible if the device current does not reach a suitably stable value allowing to discern variations induced by the biorecognition event with respect to inherent fluctuations in the device current. Consequently, a stabilization protocol must be designed and followed prior to biosensing tests.

**Figure 2:**
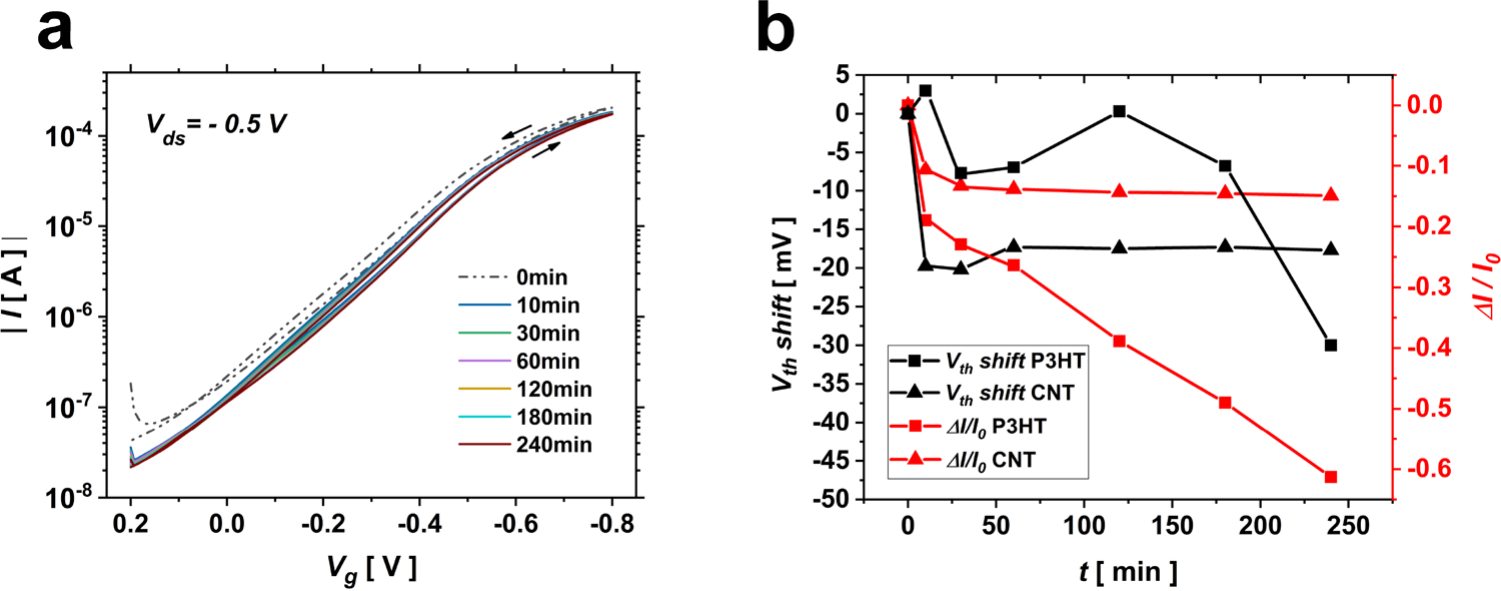
(a) Forward and backward transfer curve cycling measurements of the EG-CNTFET in DI water using a Ag/AgCl gate for a total duration of 4 h, with an interval of 3 min between successive curves; each curve acquisition lasts for 80 s. (b) Threshold voltage shift (left vertical axis) and normalized current change at −0.8 V (right vertical axis) for the EG-CNTFET (triangle symbols) and P3HT-EGOFET (square symbols) during 4 h of transfer cycling with 3 min intervals. The negative sign of the *V*_th_ shift signifies a shift toward negative potentials.

The stability of the proposed device when operating in water was evaluated using a Ag/AgCl electrode as a gate (Figure 1d) by measuring a series of consecutive transfer characteristic curves with 3 min intervals for a duration of 4 h (Figure 2a). In order to quantify any variation in device response during the test, the threshold voltage and the normalized channel current change at fixed potential (Δ*I/I_0_* at *V*_g_ = −0.8 V) were extracted from the transfer characteristic curves acquired at different time intervals. According to Figure 2a, the transfer curve undergoes the most evident change in the first ∼10 min, i.e., within the first three transfer cycles. The extracted threshold voltages (black triangle symbols in Figure 2b) reveal that after an hour of acquisition of consecutive transfer curves, the device stabilizes with a very weak residual shift of its threshold voltage of ∼1 mV/h toward negative potentials. For the purpose of comparison, an optimized inkjet-printed P3HT-based EGOFET was fabricated (details in the Supporting Information) and subjected to the same transfer cycling test, under the same biasing conditions, in DI water. As shown in Figure 2b, the threshold voltage of the P3HT-based EGOFET (black square symbols) does not stabilize after 4 h of transfer cycling. Correspondingly, the normalized current change for the EG-CNTFET (Figure 2b, red triangle symbols) shows a 13.8% decrease during the first hour of transfer cycling, before showing a substantial stabilization, with a residual very weak and constant decay of 0.002 h^–1^ until the end of the fourth hour. The P3HT-EGOFET (Figure 2b, red square symbols) shows a 26.4% decrease in normalized current change after an hour of acquisition of consecutive transfer characteristic curves. Then, the current decay exacerbates without a constant rate until the end of the fourth hour, reaching a reduction of more than 60% and indicating that the device needs a longer conditioning procedure before showing a stable response. In fact, in agreement with the previous literature,^18^ the inkjet-printed P3HT-EGOFET needs 30 h of transfer cycling before producing a stable current response with a Δ*I/I*_0_ change rate of 0.002 h^–1^ (Figure S2).

Overall, acquisition of consecutive transfer characteristic curves shows that an hour of transfer cycling in DI water is enough to stabilize the *I*–*V* response of the EG-CNTFET, with a negligible residual threshold voltage shift of 1 mV/h and Δ*I/I*_0_ decay rate of 0.002 h^–1^. This evidence shows that the device can be potentially employed for biosensing after only an hour of preconditioning and is stable for at least three more hours.

### 3.3 Biosensing Test

To assess the suitability of our EG-CNTFET as the base of a biosensor, we performed a proof-of-principle experiment aimed at sensing the biotin–streptavidin binding event. For this purpose, a gold electrode is introduced, acting as the sensing gate (F-gate): such a gate is biofunctionalized to accommodate a biotin-terminated recognition layer. Apart from the F-gate used for biosensing, a Ag/AgCl reference gate, denoted as the C-gate, is utilized to monitor the stability of the device during biosensing measurement.

**Figure 3:**
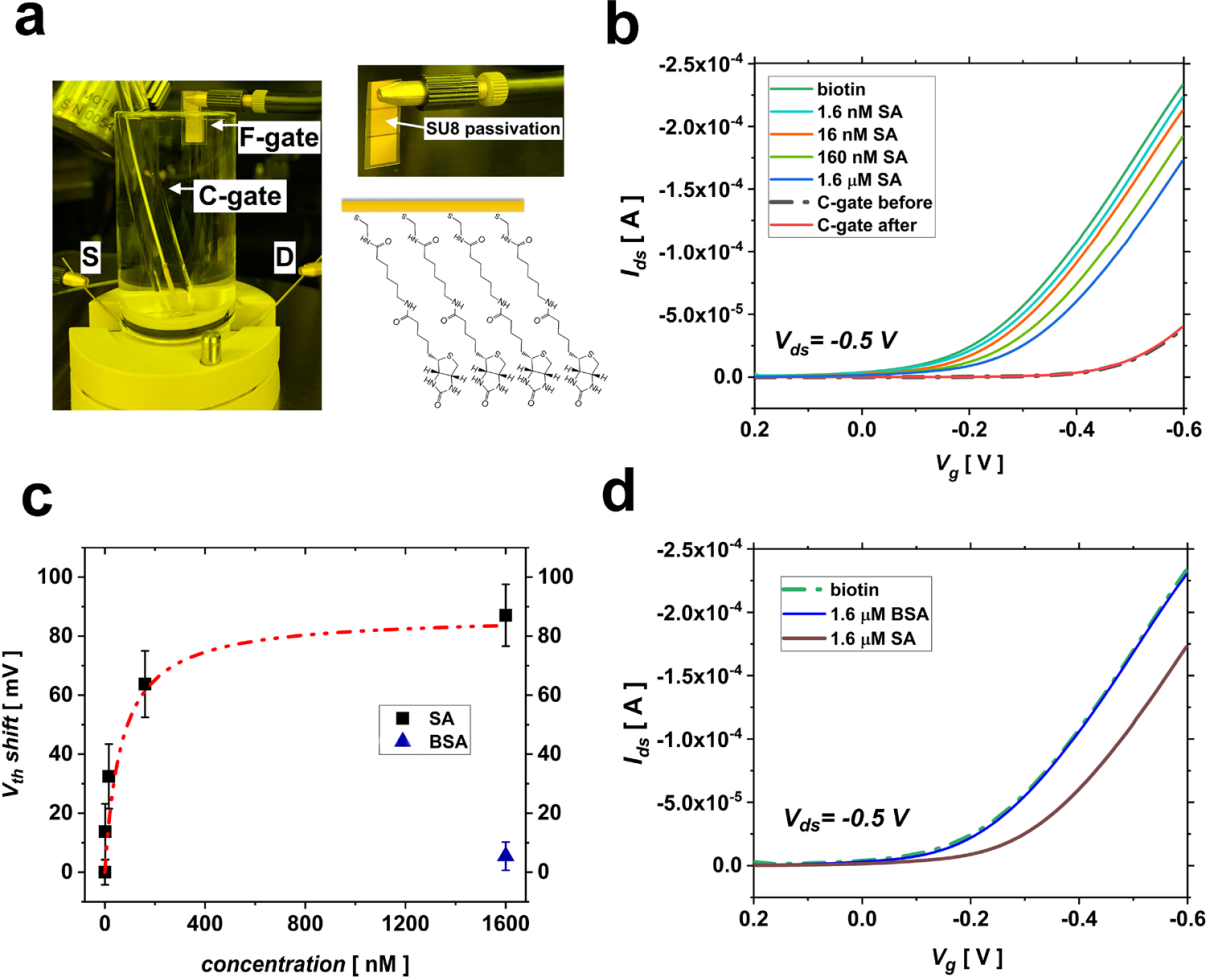
(a) Biosensing setup that uses two gates: an F-gate for biofunctionalization and a C-gate (Ag/AgCl) for controlling the stability of the channel while being exposed to DI water; top right: detail of the F-gate; bottom right: schematic illustration of the biotin-terminated SAM immobilized on the F-gate. (b) Transfer characteristic curves obtained for the F-gate exposed to different concentrations of streptavidin. (c) Calibration curves acquired for a threshold voltage shift, together with a Langmuir adsorption isotherm fit (dashed dotted line); the blue triangle symbol shows the device response to 1.6 μM BSA. (d) Negative control test comparing the transfer curves recorded for a non-exposed sensing gate denoted as “biotin” and sensing gates exposed to a 1.6 μM streptavidin solution and to a 1.6 μM BSA solution.

A schematic illustration of F-gate biofunctionalization is shown in Figure 3a. In detail, a self-assembled monolayer (SAM) of cysteamine (Cys-SAM) is grown on the gold electrode. Then, treatment of Cys-SAM-terminated gold electrodes with NHS-LC-biotin molecules results in substitution of the NHS group with the amine group of Cys-SAM. As a result, a molecular bioreceptor layer forms, anchored to the gold electrode through a thiol group at one end, exposing a biotin moiety at the other end (biotin-SAM). The biosensing experiment involves the exposure of the biotin-functionalized F-gates to standard solutions of streptavidin, with concentrations varying from 1.6 nM to 1.6 μM. The binding of streptavidin to the biotin-SAM is then sensed by using the exposed F-gates as gating electrodes of the EG-CNTFET. It should be noted that pure water is a weak electrolyte, following self-ionization ([H_3_O^+^] = [OH^–^] = 10^–7^ M) and dissolution of carbon dioxide.^39^ Therefore, its Debye length is quite long, typically 100 nm,^4^ well above the distance at which the binding occurs considering the theoretical length of the molecular receptor that is about 3 nm.

As shown in Figure 3b, exposure of the biofunctionalized F-gate to increasing concentrations of streptavidin leads to recording a correspondingly increasing negative shift in the threshold voltage. Figure 3c presents the titration curve obtained considering the device threshold voltage shift extracted at *V*_g_ = −0.5 V from the transfer curves obtained for different concentrations of the target protein. Comparing the lowest threshold voltage shift recorded in our experiment for streptavidin-captured F-gates (13.75 mV for 1.6 nM streptavidin) with the threshold voltage shift that the device experiences after being subjected to the stabilization procedure for an hour (∼1 mV/h), it is concluded that the recorded threshold shifts for different concentrations of streptavidin can solely be attributed to the biotin–streptavidin binding event taking place on the surface of F-gates and not to any instability of the channel. A threshold voltage shift of 87 mV is measured upon exposure of the sensing gate to 1.6 μM streptavidin (Figure 3c), while a 34% decrease in normalized current change is recorded for the same concentration of the target protein (Figure S3). The dose curve obtained based on the threshold voltage shift can be fitted with a Langmuir adsorption isotherm model being presented as a dashed dotted line in Figure 3c.

The selectivity of the sensor was evaluated by recording its response toward bovine serum albumin (BSA), which has a comparable molecular weight with respect to streptavidin (∼60 kDa), as biotin does not have a selective affinity to BSA. Figure 3d presents the transfer characteristic curve recorded for the biotin-immobilized F-gate in the presence of 1.6 μM BSA, compared to the one obtained for an exposure to 1.6 μM streptavidin. An average threshold voltage shift of 5.4 mV (three physically different gates where tested) is recorded in response to 1.6 μM BSA, which is negligible compared to a threshold voltage shift of 87 ± 5.7 mV obtained for 1.6 μM streptavidin. This experiment successfully rules out the possibility of non-specific absorption at the surface of the sensing gate, ensuring the device selectivity toward streptavidin.

To gain a better insight into the device potentiometric sensing mechanism, one can consider the biotin-terminated biorecognition layer immobilized on the F-gate as an ion-selective membrane.^6^ As the excess interfacial charge of the biotin-terminated layer is screened by counterions in water, an electrochemical potential drop is created, which is a quantitative function of the concentrations of the biotin, streptavidin, and resulting binding complex according to the Donnan equation:^6^
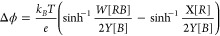
1

where *k_B_* is the Boltzmann constant and *T* is the temperature, Δϕ is the electrochemical potential drop, *R* is biotin that carries charge *X*, *B* is streptavidin that possesses charge *Y*, and *RB* is the resulting binding complex that carries charge *W*. Such a potential drop at the F-gate/electrolyte interface has to be compensated by applying a more negative *V*_g_ to achieve a given value of current, meaning that the device demonstrates a negative shift in its threshold voltage.

Our finding is consistent with previous reports where a threshold voltage shift was evidenced as the main consequence of the binding event.^4,7,8,21,38^

The extraction of the transconductance of the device for different concentrations of streptavidin (Figure S5a) shows the presence of a noticeable shift toward negative potentials due to the threshold voltage shift of the device. Maximum transconductance values (*g*_m_^max.^) for different concentrations of streptavidin were extracted from Figure S5a. By calculating the normalized maximum transconductance change (Δ*g*_m_^max.^/*g*_m_0__^max.^) for different protein concentrations, a decrease of 3.6% is recorded for a 1.6 μM concentration (Figure S5b). This is a relatively small change when compared to a 34% decrease in Δ*I/I*_0_ measured for the same concentration of the protein (Figure S3). This indicates that the overall capacitance of the device has only slightly changed, assuming the channel’s mobility to be unaffected by protein binding. This is explained by our device design characteristics where the gate surface area is 100 times larger than the active area of the device, resulting in a much larger capacitance at the gate/electrolyte interface. In this case, the drain current remains unaffected by the variation in the capacitance at the gate/electrolyte interface, where the biorecognition layer is immobilized.

Finally, the theoretical LOD was calculated from the dose curve based on the threshold voltage shift presented in Figure 3c. The LOD was measured as the concentration of streptavidin that corresponds to a response of (*V*_th_)_mean_ ± *k*σ, where (*V*_th_)_mean_ is the average value of the blank signal, σ is the standard deviation of the blank sample, and *k* is a numerical factor selected based on the confidence level needed. IUPAC suggests a value of *k =* 3 to be acceptable since the probability of a false positive would be less than 1%.^21^ The standard deviation of the blank measurement (biotin-functionalized F-gate) was calculated as 4.23 mV, which returns a theoretical LOD of 1.47 nM based on Langmuir fit (dashed dotted line) presented in Figure 3c. Comparing to an only report on sensing streptavidin by an EGOFET,^15^ our device showed 7 times lower LOD while having almost the same range.

## 4 Conclusions

We have proposed a water-gated FET based on cost-effective inkjet-printed random networks of polymer-wrapped s-SWCNTs as a candidate for future biosensors. Our EG-CNTFET requires only an hour of device conditioning in water, a much shorter time with respect to analogue polymer-based devices exploited for highly sensitive biosensors. After conditioning, the water-gated device demonstrates excellent stability, with a *V*_th_ shift of 1 mV/h and Δ*I/I*_0_ decrease rate of 0.002 h^–1^. When gated through water, the devices show a normalized transconductance (*g*_m_ divided by *W/L*) of 0.44 μS, being at least one order of magnitude higher compared to their EGOFET counterparts.

As a proof-of-principle, we also demonstrate the potential applicability of our EG-CNTFET in biosensing by characterizing the well-known biotin–streptavidin binding event through immobilization of biotin on the transistor gate. The biosensor correctly operates in the tested range of 1.6 nM to 1.6 μM of streptavidin, has an estimated LOD of 1.47 nM, and shows no response to BSA in a negative control experiment, indicating selectivity. Having validated the compatibility of the device with such a biosensing scheme, our work paves the way for the future exploration of the use of such stable and performing platforms with a plethora of recognition strategies, including protein- and genomic-based ones, and of the adoption of advanced biofunctionalization protocols, with the potential to provide a very high sensitivity.

Overall, our EG-CNTFET shares the benefits of cost-effective and large-area processing of polymer EGOFETS while being characterized by a higher normalized transconductance, a shorter conditioning time, and excellent stability to operation in water. For such reasons, we believe it is a powerful candidate for applications in the bioelectronics field, offering a reliable platform for fast response diagnostic tools.

## Supporting Information Available

Schematic representation of the biosensor; description of the fabrication procedure of P3HT-based EGOFETs; extracted current values at −0.8 V from consecutive transfer curves acquired for P3HT-based EGOFETs for 65 h; calibration curve based on normalized current change at −0.5 V for EG-CNTFETs; typical transfer curves for bare Au and Cys-SAM-functionalized Au electrodes; transconductance curves for different concentrations of streptavidin together with titration curves based on extracted maximum transconductance values; and water contact angle measurements on three physically different biotin-functionalized F-gates (PDF)
