# Supplementary material for: A Rapidly Stabilizing Water-Gated Field-Effect Transistor Based on Printed Single-Walled Carbon Nanotubes for Biosensing Applications
Source: ACS Appl Electron Mater. 2021 Jul 1;3(7):3106–13. doi: 10.1021/acsaelm.1c00332 (PMC8411763; doi:10.1021/acsaelm.1c00332)
Supplement: Supplementary file 1 — el1c00332_si_001.pdf [file el1c00332_si_001.pdf]

Supporting Information

A Rapidly Stabilizing Water-Gated Field-Effect Transistor Based on Printed Single-Walled Carbon Nanotubes for Biosensing Applications

Alireza Molazemhosseini, [†] *Fabrizio Antonio Viola,* [†] *Felix J. Berger,* [‡] *Nicolas F. Zorn,* [‡] *Jana Zaumseil,* [‡] *Mario Caironi* [†,*]

[†] Center for Nano Science and Technology @PoliMi, Istituto Italiano di Tecnologia, Via Giovanni Pascoli, 70/3, 20133 Milano, Italy

[‡] Institute for Physical Chemistry and Centre for Advanced Materials, Universität Heidelberg, D-69120 Heidelberg, Germany

[*] Corresponding Author, Email: mario.caironi@iit.it

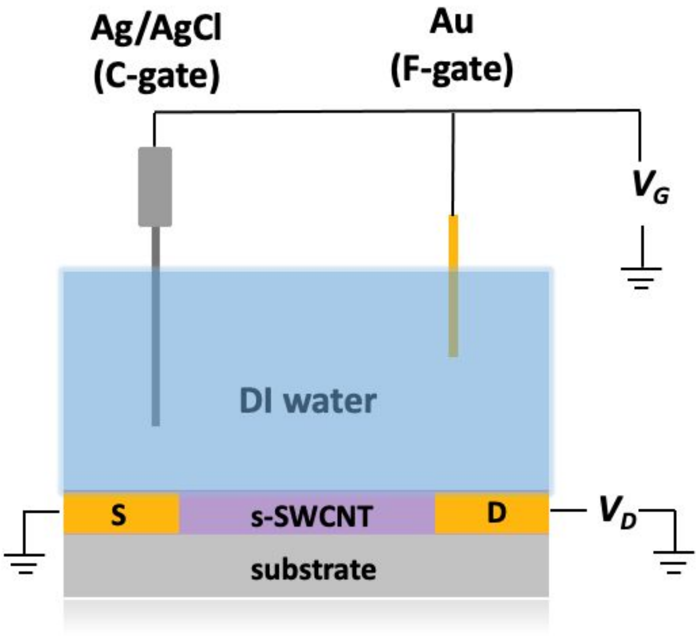

Figure S1. Schematic representation of the biosensor: it consists of an EG-CNTFET, a reference electrode (C-gate) made of Ag/AgCl to monitor the stability of the channel, and a gold gate (F-gate) for functionalization.

Fabrication of inkjet-printed P3HT based EGOFET

Poly(3-hexylthiophene) (P3HT; Sigma Aldrich, regio-regularity 99%, average MW = 20000 – 45000 g.mol^{-1}) was dissolved in 1,2-dicholorobenzene (ODCB) at a concentration of 2.6 mg.mL^{-1} and then inkjet-printed by means of a Fujifilm Dimatix onto the active area of interdigitated source-drain electrodes with channel width (*W*) of 4.4 mm and length (*L*) of 3 µm patterned on glass (the same electrodes used for EG-CNTFET). The ink was printed at a drop spacing of 45 mm. The device was annealed at 130 °C for 8 hours in an inert atmosphere.

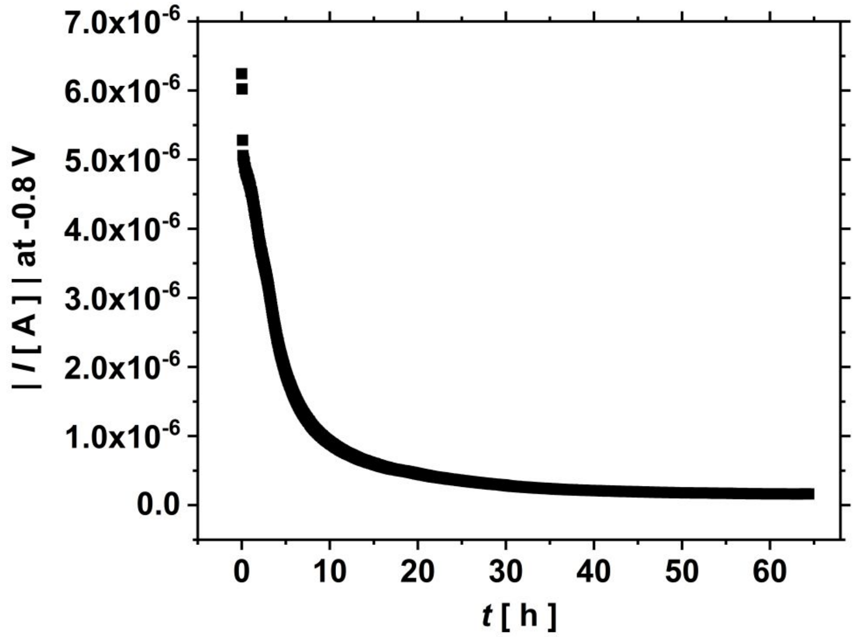

Figure S2. Current values extracted at -0.8 V from consecutive transfer curves acquired with 3 min interval for inkjet-printed P3HT based EGOFET.

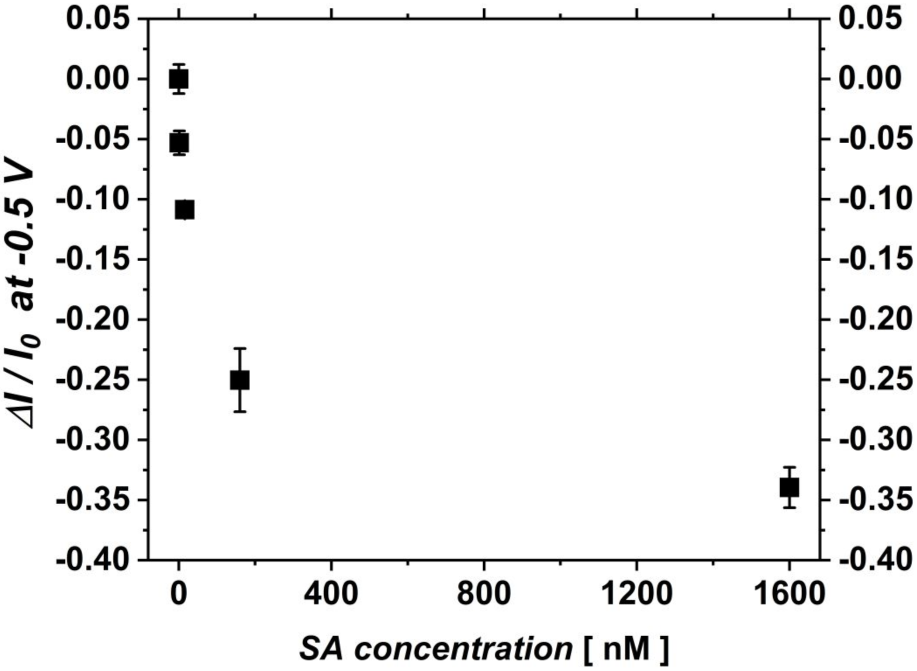

Figure S3. calibration curve based on normalized current change at -0.5 V extracted from transfer curves presented in Figure 3b.

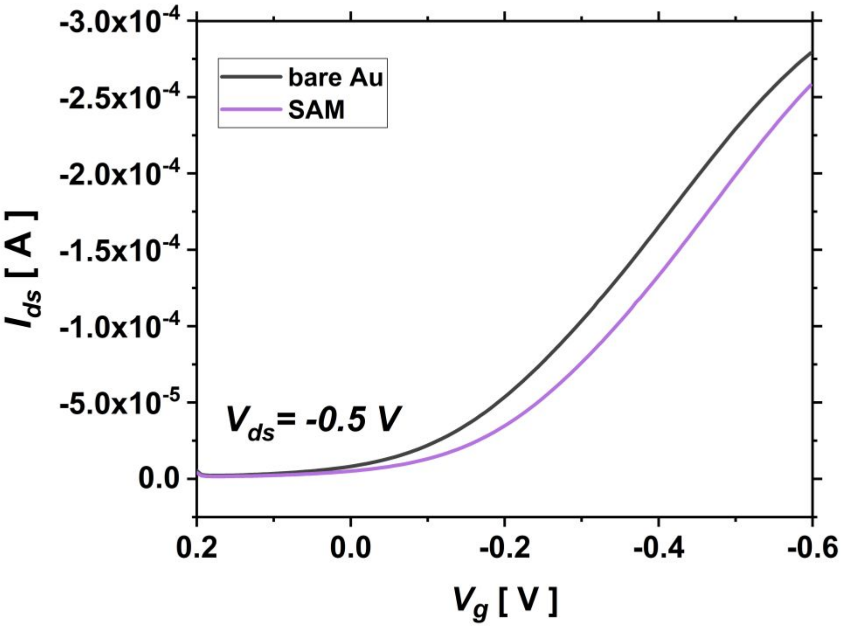

Figure S4. Typical transfer characteristic curves for a bare gold gate as well as Cys-SAM functionalized gold gate.

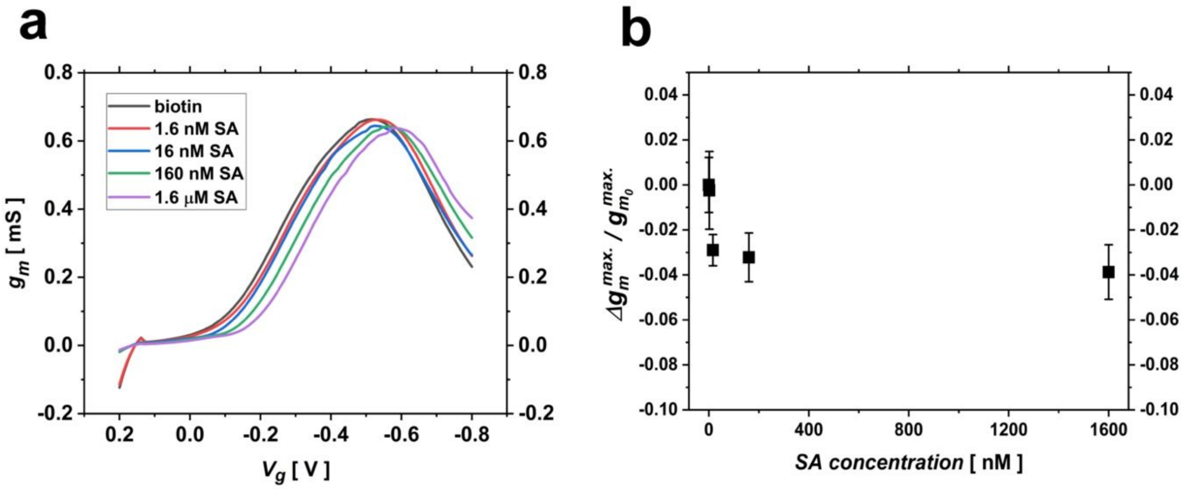

Figure S5. (a) transconductance curves for different concentrations of streptavidin extracted from transfer curves presented in Figure 3b; F-gate not exposed to streptavidin is denoted as "biotin". (b) titration curve for normalized maximum transconductance extracted from curves in (a).

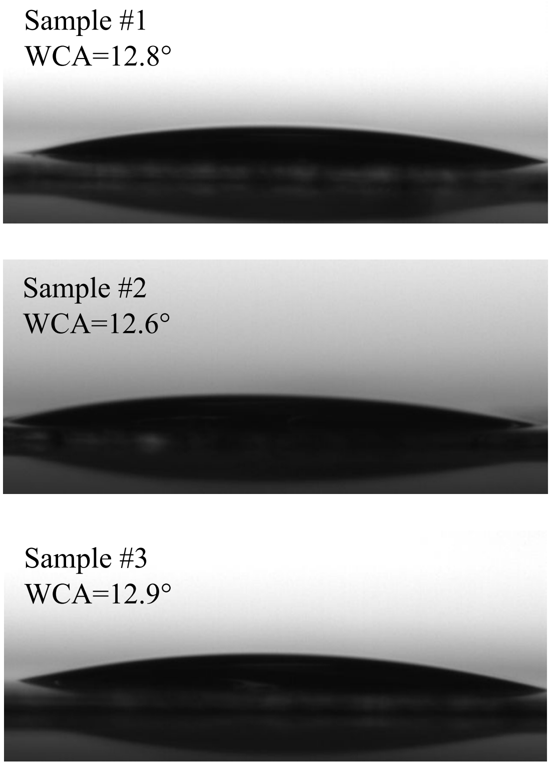

Figure S6. Water contact angle measurements on three physically different biotin functionalized F-gates in order to confirm the reproducibility of the functionalization procedure. The droplet volume is 10 μL.
